# Comparing 2-day vs 3-day flu-CY lymphodepleting regimens for CD19 CAR T-cell therapy in patients with non-hodgkin’s lymphoma

**DOI:** 10.3389/fimmu.2024.1403145

**Published:** 2024-06-14

**Authors:** David G. Frame, Marcus Geer, Salena Kasha, Denise Markstrom, Gianni Scappaticci, Tate Feeney, Andrew Hayduk, Hilary M. Mansoor, Avery Oberfeld, Hannah D’Antonio, Sarah Anand, Sung Won Choi, John Maciejewski, Attaphol Pawarode, Mary Mansour Riwes, Muneesh Tewari, John Magenau, Monalisa Ghosh

**Affiliations:** ^1^ Department of Clinical Pharmacy, College of Pharmacy, University of Michigan, Ann Arbor, MI, United States; ^2^ Department of Internal Medicine, College of Medicine, University of Michigan, Ann Arbor, MI, United States; ^3^ School of Pharmacy, College of Pharmacy, University of Michigan, Ann Arbor, MI, United States; ^4^ Department of Pharmacy Services and Clinical Pharmacy, Michigan Medicine, Ann Arbor, MI, United States; ^5^ Department of Pediatrics, Michigan Medicine, University of Michigan, Ann Arbor, MI, United States

**Keywords:** CAR T-cell, non-hodgkin’s lymphoma, lymphodepletion, CRS, ICANS

## Abstract

**Introduction:**

Lymphodepleting chemotherapy (LDC) is critical to CAR T-cell expansion and efficacy. Despite this, there is not a consensus in the literature regarding the optimal LDC regimen, including dose and frequency.

**Methods:**

We retrospectively reviewed consecutive patients at a single institution that received LDC prior to treatment with the CD19 directed CAR T-cell products axicabtagene ciloleucel and tisagenlecleucel. Patients treated at our center received fludarabine 30 mg/m^2^ and cyclophosphamide 500 mg/m^2^ for 3 consecutive days prior to May 2019. After this timepoint patients routinely received fludarabine 40 mg/m^2^ and cyclophosphamide 500 mg/m^2^ for 2 consecutive days. Clinical data from each cohort were obtained from the electronic medical record and compared for differences in CAR T-cell efficacy and toxicity.

**Results:**

From June 2018 to August 2023, LDC was given to 92 patients prior to CD19 directed CAR T-cell therapy for relapsed non-Hodgkin’s lymphoma. Twenty-eight patients received a 3-day regimen, and 64 patients received a 2-day regimen. In the total cohort, 75% of patients received axicabtagene ciloleucel and 25% received tisagenlecleucel. The overall response rates in both the 2-day regimen group and the 3-day regimen group were similar (69% vs 75%, p= 0.21) as were the complete response rates (50% vs 54%, p=0.82). There were no significant differences between the 2-day and 3-day regimens for grade 2–4 cytokine release syndrome (55% vs 50%, p=0.82), grade 2–4 immune effector cell associated-neurotoxicity syndrome (42% vs 29%, p=0.25), or time to resolution of neutropenia or thrombocytopenia. The rate of prolonged platelet recovery lasting greater than 60 days was higher with the 3-day regimen (9% vs 27%, p=0.026).

**Discussion:**

As the number of patients eligible for CAR T-cell therapy continues to increase, optimizing each component of therapy is necessary. We show that a 2-day regimen of LDC with fludarabine and cyclophosphamide is feasible without significant impact on CAR T-cell efficacy or toxicity. Prospective studies are necessary to further determine the most effective LDC regimen.

## 1 Introduction

Chimeric antigen receptor (CAR) T-cell therapy that targets CD19 has shown efficacy in patients with relapsed and refractory large B-cell lymphoma (LBCL). Lymphodepleting chemotherapy (LDC) prior to cell infusion is critical to the adoptive cell therapy process, however there is relatively little prospective data dedicated to determining the optimal regimen (1). LDC can impact both short and long-term CAR T-cell efficacy by creating a favorable immune environment for *in vivo* product expansion and by promoting the formation of memory T-cells (2, 3). A more favorable immune environment is created through several mechanisms including the activation and recruitment of innate immune cells, increased availability of the cytokine pool, and reduction in the number of immunoregulatory T-cells (1). Despite the importance of these factors, there is significant heterogeneity among the LDC regimens reported in the literature. A recent review of 1271 publications on CAR T-cell therapy for LBCL found that fludarabine and cyclophosphamide were the most common drugs used, however, the cumulative dose varied from a total dose of 75–120 mg/m^2^ for fludarabine and 750–1500 mg/m^2^ for cyclophosphamide (1). The pivotal ZUMA-1 trial that led to the FDA approval of axicabtagene ciloleucel (axi-cel, Kite Pharma, Inc. Santa Monica, CA) in LBCL used fludarabine 30 mg/m^2^ and cyclophosphamide 500 mg/m^2^ for 3 days (4). The TRANSCEND and TRANSFORM registration studies that led to the FDA approval of lisocabtagene (liso-cel, Bristol-Myers Squibb, Bothell, WA) used fludarabine 30 mg/m^2^ and cyclophosphamide 300 mg/m^2^ for 3 days (5, 6). The ELIANA and JULIET registry studies led to the FDA approval of tisagenlecleucel (tisa-cel, Novartis Pharmaceuticals Corporation, East Hanover, NJ) and used fludarabine 25 mg/m^2^ and cyclophosphamide 250 mg/m^2^ for 3 days (7, 8). It is unclear how much the differences in outcomes between these products, if any, can be attributed to the differences in LDC dosing.

Variations in LDC exposure may influence CAR T-cell expansion and toxicity (9, 10). Higher cumulative doses of LDC have been shown to improve the depth of lymphodepletion as well as disease response after CD19 CAR T-cell therapy (10, 11). Using a fludarabine population pharmacokinetics model, it was reported that patients with low fludarabine exposure had a higher risk of relapse (9). Conversely, relatively high fludarabine exposure increased the risk of developing immune effector cell-associated neurotoxicity syndrome (ICANS). Fludarabine also has the potential to affect the hematopoietic stem cell (HSC) compartment which could play an initial role in long term myelosuppression (11). However, it is not clear if the fludarabine dose contributes to the risk for long-term cytopenias that are often seen in this patient population.

As the indications for CAR T-cell therapy have expanded rapidly, the logistical burden to providing care has also increased at a rapid pace. Due to resource constraints, many treating institutions are offering CAR T-cells as a primarily outpatient therapy to minimize hospital bed utilization. To better meet these evolving needs and to decrease the burden on patients, our center transitioned from a 3-day LDC regimen of fludarabine 30 mg/m^2^ and cyclophosphamide 500mg/m^2^ on days -4, -3, and -2 to a 2-day LDC regimen of fludarabine 40mg/m^2^ and cyclophosphamide 500mg/m^2^ on days -3 and -2 prior to CAR T-cell infusion. Here we report the outcomes of patients with non-Hodgkin lymphoma (NHL) who received this abbreviated LDC regimen prior to infusion of commercial axi-cel or tisa-cel.

## 2 Methods

### 2.1 Study design

This study is a single-center retrospective review of consecutive patients with NHL who received commercial axi-cel or tisa-cel and a fludarabine/cyclophosphamide-based LDC regimen at our center between June 2018 and August 2023. The study was approved by the University of Michigan institutional review board and all data was abstracted directly from the electronic medical record. All patients were included for safety and response analysis. Response to CAR T-cell therapy was primarily determined by positron emission tomography (PET) scan using Lugano criteria unless there was evidence of disease progression based upon clinical findings or biopsy (12). Per institutional protocol, PET scans were obtained approximately 8 weeks following CAR-T infusion and then every 3 months for the first year. Overall response rate (ORR) was defined as the percentage of patients who achieved a partial response (PR) or better after CAR-T infusion. Progression-free survival (PFS) was defined as the time from CAR T-cell infusion until relapse, progression, or death from any cause. Overall survival (OS) was defined as the time from CAR T-cell infusion until death of any cause.

Infections were recorded for all patients for the first 30 days after CAR-T infusion and for 6 months in patients who had a complete response (CR) to therapy. Cytopenias were assessed at 30 days following CAR T-cell infusion in all patients and prolonged cytopenias beyond 60 days following CAR T-cell infusion were assessed in patients who had a CR or near CR on the 8-week PET scan to avoid any effects of disease or subsequent chemotherapy on prolonged myelosuppression. Neutrophil recovery was defined as an absolute neutrophil count (ANC) > 1000 cells/mm^3^ without growth factor support on consecutive measurements. Platelet recovery was defined as the day platelets were consistently greater than 50,000 cells/mm^3^ without growth factor support.

Fludarabine exposure (area under the curve [AUC]) for each patient was calculated retrospectively using a population PK model as previously published using the Cockroft-Gault equation with actual height and weight (13).

### 2.2 Patient management

All patients received LDC using either fludarabine 40mg/m^2^ and cyclophosphamide 500mg/m^2^ on days -3 and -2 prior to CAR T-cell infusion (2-day regimen) or fludarabine 30mg/m^2^ and cyclophosphamide 500mg/m^2^ on days -4, -3, and -2 prior to CAR T-cell infusion (3-day regimen). The 2-day regimen was based on literature demonstrating significantly improved CAR T-cell expansion and efficacy with the addition of fludarabine to cyclophosphamide relative to single-agent cyclophosphamide or cyclophosphamide/etoposide (14). Similarly, the literature supports the importance of the fludarabine AUC and the fludarabine AUC in the 2-day vs 3-day regimens was estimated to be equivalent (9). The LDC was administered primarily in the outpatient setting. Patients were subsequently admitted to the hospital prior to cell infusion for observation. Grading of cytokine release syndrome (CRS) and ICANS followed the American Society for Transplantation and Cellular Therapy (ASTCT) recommendations (15). Management of CRS and ICANS was at the discretion of the attending physician at the time of the event. Institutional guidelines, which did not change during the time of this study, outline the use of tocilizumab for CRS grade ≥2 as well as persistent grade 1 CRS. Corticosteroids and/or siltuximab were second line for CRS. Corticosteroids were the first line of treatment for ICANS. Additional agents for severe/refractory ICANS included siltuximab, anakinra, and intrathecal steroids. Seizure prophylaxis with levetiracetam was given to all patients for 8-weeks following CAR T-cell infusion. All patients received antimicrobial prophylaxis starting the day of CAR T-cell infusion including viral, bacterial, and fungal prophylaxis. Prior to July 2019, all patients received an extended spectrum azole for antifungal prophylaxis. However, after July 2019 fluconazole was used as the initial agent unless patients had prolonged neutropenia either before or after CAR T cell infusion, or prior fungal infection. Use of granulocyte colony stimulating factors was restricted until after day 21 post CAR-T infusion. Patients were treated for neutropenic fever according to institutional protocol.

### 2.3 Statistical methods

Descriptive statistics included mean, standard deviation, median, and range for continuous variables, and percentages for categorical variables. The association between variables was calculated using Fisher’s exact test or Chi-squared test. Comparability of the two cohorts was evaluated with t-test or Mann-Whitney test. PFS and OS were estimated by the Kaplan-Meier method and log-rank test was used to evaluate the difference in PFS or OS between patient groups. Patients that were alive without disease relapse were censored at the date of last follow-up.

## 3 Results

A total of 92 patients received LDC prior to infusion of commercial axi-cel or tisa-cel between June 2018 and August 2023. Sixty-four patients (69.6%) received the 2-day LDC regimen and 28 (30.4%) received the 3-day regimen. Patient and disease characteristics are summarized in **Table 1**. There were more lines of prior therapy in the 3-day group (median: 4 vs 3, p=0.010) and more frequent use of bridging therapies (72% vs 39%, p=0.0048) in the 2-day group (**Table 2**). There was a numerically greater use of axi-cel (81% vs 61%) in the 2-day group that did not reach statistical significance. The most frequent indication for therapy in both cohorts was diffuse large B-cell lymphoma, not otherwise specified (DLBCL NOS) followed by transformed follicular lymphoma in the 3-day group and high-grade B-cell lymphoma in the 2-day group. The cell-of-origin differed between groups (p=0.041). The 3-day cohort included more germinal center (GC) (55% vs 75%) and the 2-day included more non-GC (33% vs 14%) B-cell phenotypes. The median follow-up time from infusion to death or last visit was 12.8 months (0.6 - 48.5) for patients receiving the 2-day regimen and 19.1 months (1.4–67.5) for the 3-day regimen.

**Table 1:** Patient demographics.

Characteristics	2-Day LDC Regimen (n=64)	3-Day LDC Regimen (n=28)	p-value
Age, years median (range)	63 (29–78)	65 (42–75)	0.189
Sex, N (%) Male	39 (61)	19 (68)	0.151
Disease Stage, N (%) I-II III-IV unknown	12 (19) 50 (78) 2 (3)	7 (25) 19 (69) 2 (7)	0.571
Histology N (%) DLBCL NOS HGBCL Transformed FL FL	31 (48) 15 (23) 11 (17) 7 (11)	15 (54) 1 (4) 7 (25) 5 (19)	0.821
Cell of Origin, N(%) GCB Non-GCB unknown	35 (55) 21 (33) 8 (13)	21 (75) 4 (14) 3 (11)	0.041
Previous Lines, median (range)	3 (1–7)	4 (2–7)	0.010
Bridging Therapy, N(%)	46 (72)	11 (39)	0.0048
Prior autologous HSCT, N(%)	8 (13)	5 (18)	0.519
CAR T-cell Product Axi-cel	52 (81)	17 (61)	0.065
Tisa-cel	12 (19)	11 (39)	

LDC, Lymphodepleting chemotherapy; DLBCL NOS, diffuse large B-cell lymphoma not otherwise specified; HGBCL, high-grade B-cell lymphoma; FL, follicular lymphoma; GCB, germinal center B-cell; HSCT, hematopoietic stem cell transplant; axi-cel, axicabtagene ciloleucel; tisa-cel, tisagenlecleucel.

**Table 2:** Bridging therapies.

	2-day LDC Regimen (n=64); N (%)	3-day LDC Regimen (n=28); N (%)	All Patients (n=92); N (%)
None	18 (28)	17 (61)	35 (38)
Any Bridging	46 (72)	11 (39)	57 (42)
Radiation	8 (12.5)	5 (18)	13 (14)
Platinum regimens*	10 (15.6)	4 (20)	13 (14)
Polatuzumab +Rituximab**	20 (31.2)	0	20 (22)
Glucocorticoids	4 (6.3)	1 (4)	5 (5.4)
Other	4 (6.3)	1 (4)	6 (6.5)

*Platinum containing regimens: rituximab/ifosfamide/carboplatin/etoposide; rituximab/gemcitabine/oxaliplatin; rituximab/dexamethasone/cytarabine/cisplatin; rituximab/dexamethasone/cytarabine/oxaliplatin.

** +/- Bendamustine.

LDC: lymphodepleting chemotherapy.

### 3.1 Response

**Figure 1:**
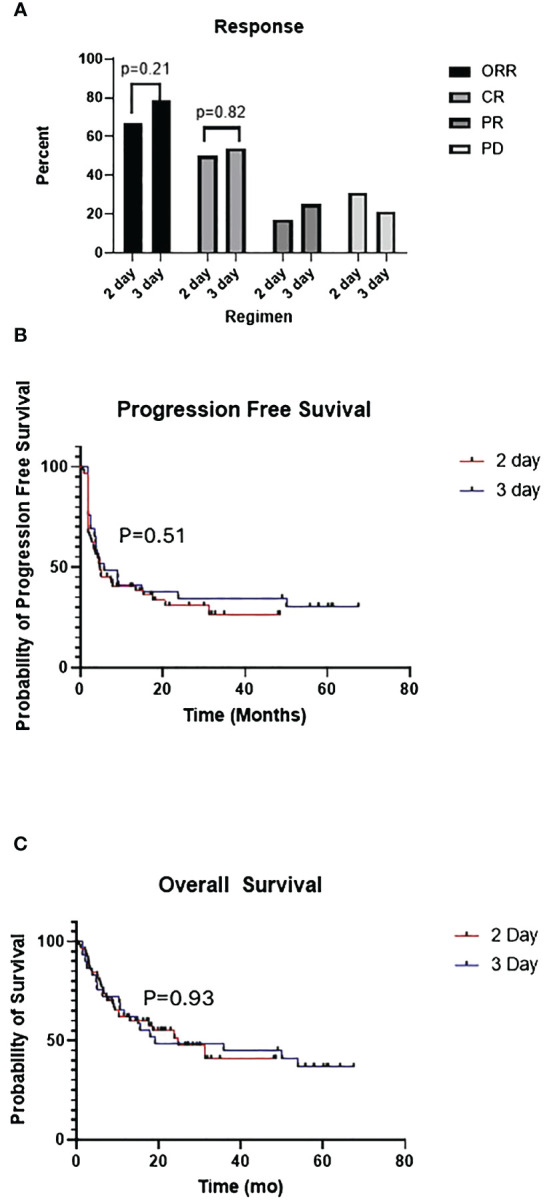
**(A)** Comparison of overall response (ORR), complete response (CR), partial response (PR), progressive disease (PD) between 2-day and 3-day lymphodepleting chemotherapy (LDC) regimens. **(B)** Kaplan-Meier curves for progression free survival (PFS) of 2-day vs 3-day LDC regimens. **(C)** Kaplan-Meier curves for overall survival (OS) of 2-day vs 3-day LDC regimens.

The overall response rate (CR + PR) and CR rate were similar between the 2-day and 3-day regimens: 69% vs 75% (p=0.21) and 50% vs 54% (p=0.82) respectively. The median progression free survival was 4.8 vs 5.8 months (p=0.506) in the 2-day vs 3-day cohorts, and the median overall survival was 24.8 and 19.1 months (p=0.925) respectively (**Figure 1**). Across both LDC regimens, the ORR for patients that received bridging therapy was 68% vs 83% for those that did not (p=0.431). The CR rate for patients that were bridged was 54% vs 65% for those that were not (p=0.255).

### 3.2 Lymphodepletion, CAR T-cell expansion and fludarabine AUC

To evaluate the extent of lymphodepletion, the absolute lymphocyte count (ALC) in the peripheral blood was recorded prior to LDC and on day 0 before the infusion of the CAR T-cells. Overall, the distribution of the ALC was similar between the two groups with the 2-day regimen having complete depletion of lymphocytes in 61% of patients as compared to 57% of patients with the 3-day regimen (**Table 3**). Four patients in the 2-day LDC group had an ALC greater than 0.2 cells/cmm (range 0.4–0.9) on the day of cell infusion vs 2 patients in the 3-day LDC group (range 0.3–0.8).

**Table 3:** Depth of Lymphodepletion and Cytopenias.

ALC (cells/cmm) on Day 0	2-Day LDC Regimen N (%)	3-Day LDC Regimen N (%)
0	39 (61)	16 (57)
0.1	19 (30)	8 (29)
0.2	2 (3)	2 (7)
>0.2	4 (6)	2 (7)
Hemoglobin Nadir (g/dL)		
<7	15 (23)	8 (29)
7–7.9	12 (19)	7 (25)
8–9	14 (22)	6 (21)
9–9.9	9 (14)	0
10–10.9	9 (14)	2 (7)
11–11.9	4 (6)	2 (7)
>12	1 (2)	3 (11)
Platelet Nadir (K/uL)		
<10	8 (12)	3 (11)
10–20	7 (10)	3 (11)
20–30	3 (4)	2 (7)
30–40	3 (4)	2 (7)
40–50	4 (6)	2 (7)
50–100	18 (28)	10 (36)
>100	21 (33)	6 (21)

ALC, absolute lymphocyte count; LDC, lymphodepleting chemotherapy; cmm, cubic millimeter.

**Figure 2:**
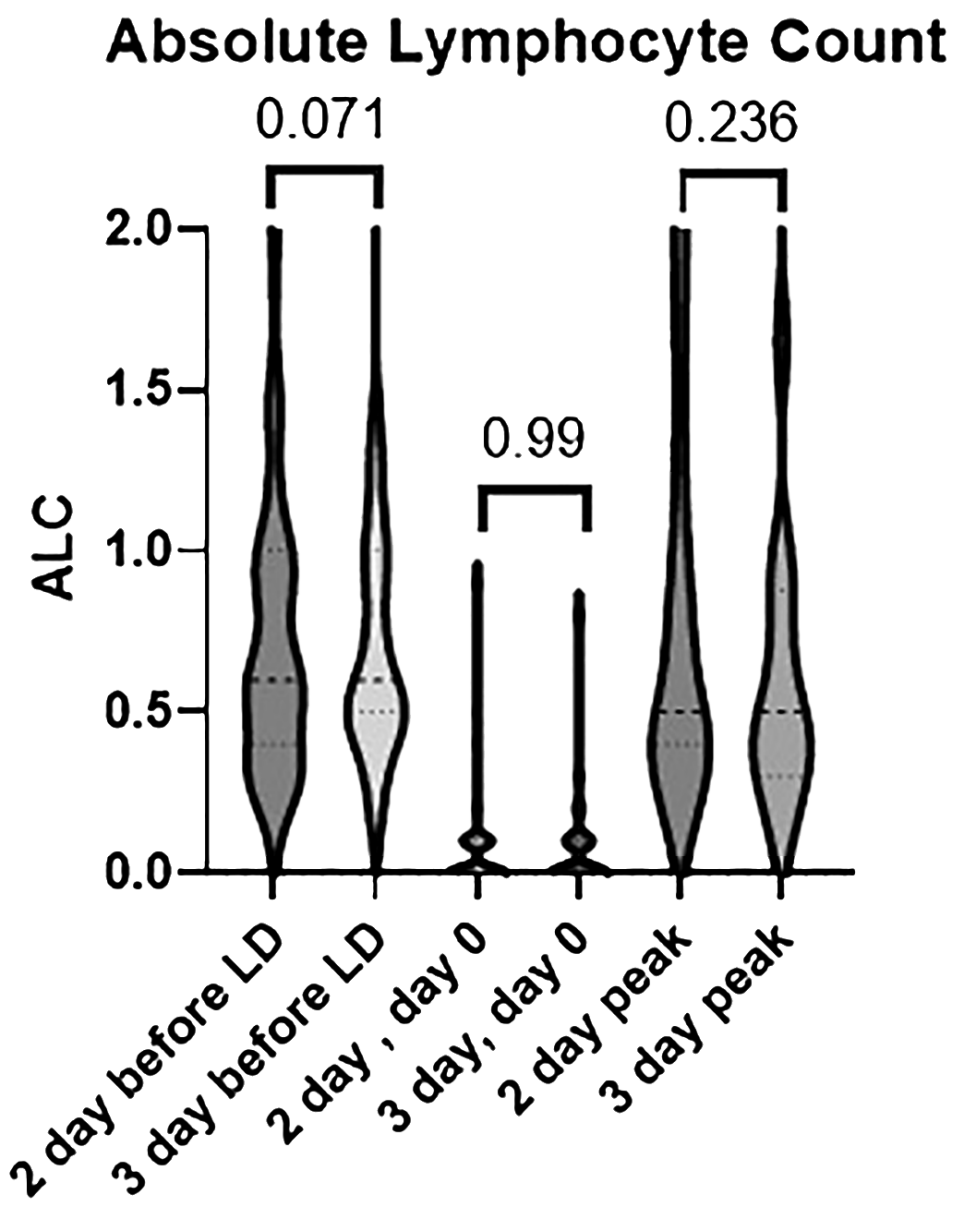
Differences in absolute lymphocyte count between the 2-day and 3-day lymphodepleting chemotherapy (LDC) regimens prior to LDC, on day 0 prior to CAR T-cell infusion, and at peak expansion post cell-infusion.

CAR T-cell expansion was not directly measured, but as a surrogate the peak ALC was measured through the first 2 weeks after CAR T-cell infusion (**Figure 2**). The median peak ALC was 0.5 cells/cmm in both regimens. The range was 0.1–2.8 with the 2-day regimen and 0.1–3 with the 3-day regimen. Overall, more patients in the 2-day regimen had a peak ALC greater than or equal to 1 and greater than 2 (31% vs 14% and 9% vs 4%, respectively).

The median estimated fludarabine exposure as measured by the area under the curve (AUC) was 19 mg*h/L in the 2-day regimen (range 8–28) and 20 mg*h/L in the 3-day regimen (range 11–35)(p=0.327). Overall, 25% and 24% (p=0.794) of patients in the 2-day and 3-day regimens respectively were within the optimal range of 18–20 mg*h/L as defined in a prior study in NHL (9).

### 3.3 Safety

#### 3.3.1 Cytokine release syndrome and immune effector cell-associated neurotoxicity syndrome

**Figure 3:**
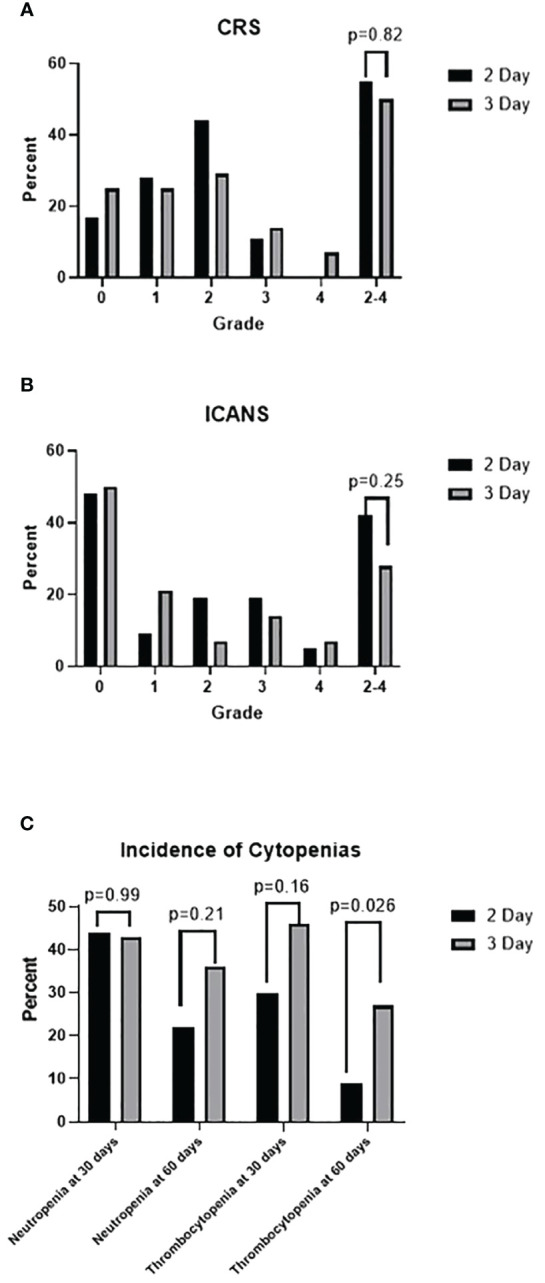
**(A)** Incidence of cytokine release syndrome (CRS) by grade. **(B)** Incidence of immune effector cell-associated neurotoxicity syndrome by grade. **(C)** Incidence of neutropenia (absolute neutrophil count < 1000 cells/mm^3^) and thrombocytopenia (platelet < 50 K/uL)at 30 days in all patients and at 60 days in patients in CR.

CRS of any grade occurred in 80% of all patients, 83% of the 2-day LDC cohort, and 75% of the 3-day LDC cohort (p= 0.41). Any-grade ICANS occurred in 51% of all patients, 53% of the 2-day LDC cohort and 50% of the 3-day LDC cohort (p=0.824). The occurrence of grade 2–4 CRS was similar between the 2-day and 3-day regimens (55% vs 50%, p=0.82), however no grade 4 CRS occurred with the 2-day regimen (**Figure 3**). There was a trend towards more frequent occurrence of grade 2–4 ICANS with the 2-day LDC regimen (42% vs 29%, p=0.25), however the difference was most evident in the amount of grade 2 ICANS (p=0.17). There was no significant difference in the incidence of grade 3 or 4 ICANS.

#### 3.3.2 Cytopenias

Hemoglobin and platelet nadirs following LDC are described in **Table 3**. Rates of hemoglobin <7 g/dL, the standard transfusion threshold for inpatient care, were 23% in the 2-day LDC cohort and 29% in the 3-day LDC cohort. Similarly, a nadir platelet count of <10 K/uL occurred in 12% of patients in the 2-day LDC and 11% of 3-day LDC.

There was no statistical difference in neutrophil or platelet recovery between the two regimens. However, the number of patients with platelet recovery by 1 month post cell infusion was greater with the 2-day regimen (70% vs 54%, p= 0.155). Thirty-two patients in the 2-day LDC cohort and 15 patients in the 3-day LDC regimen had a CR or near CR at 60 days post CAR T-cells and were evaluated for prolonged cytopenias. Fewer patients had prolonged platelet recovery of greater than 60 days (9% vs 27%, p=0.026) within the 2-day regimen with a mean recovery time of 94 days vs 137 days.

#### 3.3.3 Infections

Infections were reported if they occurred in the first 30 days after CAR-T infusion to isolate any potential differences associated with the intensity of the chemotherapy. There were no significant differences in the incidence of infections with 16 infections occurring in 15 patients who received the 2-day regimen and 10 infections occurring in 8 patients who received the 3-day regimen (**Table 4**). Notably, all the patients that received the 3-day regimen were treated before the onset of the COVID-19 pandemic. There were more *Clostridium difficile* infections in the 2-day group. This may be due to an institutional policy of screening for *C. difficile* on admission prior to cell administration, which was paused during the COVID-19 pandemic.

**Table 4:** Infections within first 30 days.

	2-Day LDC Regimen (n=64) N (%)	3-Day LDC Regimen (n=28) N (%)	p-value
Patients with any infection	15 (23%)	8 (29%)	0.609
Bacteremia	4 (6.3%)	5 (17.9%)	
Urinary tract infection	1 (1.6%)	2 (7.1%)	
Viral infections CMV viremia° Adenovirus COVID-19 Other respiratory virus	7 (10.9%) 3 (4.7%) 1 (1.6%) 2 (3.1%) 1 (1.6%)	2 (7.1%) 2 (7.1%) 0 0 0	
*Clostridium difficile* infection	4 (6.3%)	0	
Fungal infection	0	1* (3.6%)	

LDC, Lymphodepleting chemotherapy; CMV, cytomegalovirus.

°CMV viremia requiring anti-viral therapy.

*Aspergillus infection.

### 3.4 Products

Although limited by small cohorts, response and toxicity were compared within the individual CAR T-cell products to better isolate the influence of the LDC regimen (**Table 5**). The rates of CR were similar between the 2-day and 3-day groups for axi-cel (50% vs 53%; p=0.99) and tisa-cel (50% vs 55%; p=0.99). There were also no significant differences in the incidence of all-grade CRS, grade 2–4 CRS, all-grade ICANS, and grade 2–4 ICANS. Given the lack of difference between the 2-day and 3-day LDC regimens, the pooled axi-cel and tisa-cel cohorts were also compared. The ORR for all patients receiving axi-cel was 68% and 78% for tisa-cel (p=0.91). The CR rate for axi-cel was 51% and 52% for tisa-cel (p=0.99). More any-grade CRS was noted with axi-cel in the entire population (87% vs 61%, p= 0.0127) but not grade 2–4 CRS (57% vs 43% p=0.338). Any-grade ICANS was more frequent with axi-cel than tisa-cel but did not reach statistical significance (57% vs 39%; p=0.159). Grade 2–4 ICANS occurred in 43% of patients receiving axi-cel and 22% of tisa-cel (p=0.0835).

**Table 5:** Outcomes by CAR T-cell product.

	2-Day LDC, Axi-cel N: 52 (%)	3-Day LDC, Axi-cel N: 17 (%)	p-value	2-Day LDC, Tisa-cel N: 12 (%)	3-Day LDC, Tisa-cel N:11 (%)	p-value
ORR	34 (65)	13 (76)	0.551	10 (83)	8 (73)	0.640
CR	26 (50)	9 (53)	0.99	6 (50)	6 (55)	0.99
CRS, Any Grade	44 (85)	16 (94)	0.435	9 (75)	5 (45)	0.214
CRS, Grade 2–4	28 (54)	11(65)	0.575	7 (58)	3 (27)	0.214
ICANS, Any Grade	28 (54)	11 (65)	0.575	6 (50)	3 (27)_	0.40
ICANS, Grade 2–4	23 (44)	7 (41)	0.99	4 (33)	1 (9)	0.575

ORR, Overall Response Rate (Partial Response + Complete Response); CR, Complete Response; CRS, Cytokine release syndrome; ICANS, Immune effector cell associated neurotoxicity syndrome; Axi-cel, axicabtagene ciloleucel; Tisa-cel, Tisagenlecleucel; LDC, lymphodepleting chemotherapy.

## 4 Discussion

While LDC is critical for effective CAR T-cell therapy, prospective studies to maximize both efficacy and efficiency of this regimen are limited. For patients receiving commercial therapy with axi-cel or tisa-cel for non-Hodgkin’s lymphoma, a common LDC regimen is a 3-day regimen of fludarabine and cyclophosphamide per the licensing trials. As the use of CAR T-cell therapy has expanded rapidly, treatment centers must maximize resource utilization. Many institutions are meeting the rising demand for CAR T-cell infusion by moving parts of the treatment into the outpatient setting. It has been shown that outpatient administration of CAR T-cell therapy increases quality of life and has similar safety and efficacy to inpatient administration (16–18). Outpatient CAR T-cell therapy can also improve cost savings by 2-to-4-fold (19). One area which can be maximized further is the potential consolidation of the LDC regimen. Our center utilized a similar 3-day fludarabine and cyclophosphamide regimen. To maximize efficiency and facilitate moving therapy to the outpatient setting, a shift was made to a 2-day regimen that would maintain fludarabine AUC. Here we demonstrate that there were no significant differences in outcomes or safety between the regimens.

The primary limitation of this study is the inability to control for numerous competing factors which may impact CAR T-cell associated outcomes aside from lymphodepleting chemotherapy. There were notable differences in characteristics between the cohorts. The 2-day cohort included more patients with a non-GCB histology, which likely influenced the more frequent use of bridging therapy. There were also differences in bridging therapies between the groups which reflects an evolution in practice patterns and available therapy options given the limited use of polatuzumab in the earlier, 3-day LDC group. The reported effects of bridging therapy in the available literature have been mixed and may be confounded by high-risk patients preferentially receiving bridging therapy (20, 21). Despite having more aggressive histology and more bridging therapy in the 2-day cohort there were no differences in overall response, complete response, survival rates, or toxicities between these regimens. The 2-day cohort also had fewer median prior lines of therapy (3 vs 4) and a greater proportion of patients receiving axi-cel, reflecting the interval approval of axi-cel as second-line therapy. Although they have not been prospectively compared, differences in response rates and toxicity have been reported between the products. The small patient subgroups limit extensive comparison, however, there were no appreciable differences in efficacy or toxicity outcomes between the 2-day and 3-day LDC regimens for either product. This also suggests that further studies evaluating tisa-cel and axi-cel with this regimen may be beneficial.

Since fludarabine exposure has been related to response, we evaluated this using the same published fludarabine population pharmacokinetics model and did not observe a difference in the optimal median predicted fludarabine AUC in the 2-day cohort compared to the 3-day cohort (9, 13). While the median fludarabine AUC was in the optimal reported range for both groups, there were only 25% of patients that fell into this range. The percentage above or below this range was also similar between the cohorts. Ideally, this suggests that future regimens would be designed to prospectively adjust fludarabine doses per patient to potentially achieve better outcomes. The response rates and safety evaluations are similar to those reported in pivotal trials as well as in real world reports for axi-cel and tisa-cel.

This study highlights the need for dedicated prospective studies to determine an optimal regimen and minimize patient exposure to unnecessary chemotherapy. Experience has also been reported for bendamustine as an alternative fludarabine-sparing regimen that may incur less toxicities (22, 23). Within the limitations of a single center retrospective study, it appears that fludarabine 40mg/m^2^ and cyclophosphamide 500 mg/m^2^ on days -3 and -2 prior to CAR T-cell infusion is safe and efficacious while conserving resources.

## Data availability statement

The raw data supporting the conclusions of this article will be made available by the authors, without undue reservation.

## Ethics statement

The studies involving humans were approved by Institutional Review Boards of the University of Michigan Medical Campus. The studies were conducted in accordance with the local legislation and institutional requirements. Written informed consent for participation was not required from the participants or the participants’ legal guardians/next of kin in accordance with the national legislation and institutional requirements.

## Author contributions

DF: Conceptualization, Data curation, Formal analysis, Writing – original draft, Writing – review & editing. MaG: Conceptualization, Formal analysis, Investigation, Methodology, Project administration, Writing – original draft, Writing – review & editing. SK: Data curation, Formal analysis, Investigation, Writing – review & editing. DM: Data curation, Formal analysis, Investigation, Writing – review & editing. GS: Data curation, Formal analysis, Investigation, Writing – review & editing. TF: Data curation, Formal analysis, Investigation, Writing – review & editing. AH: Data curation, Formal analysis, Investigation, Writing – original draft. HM: Data curation, Formal analysis, Investigation, Writing – original draft. AO: Data curation, Formal analysis, Investigation, Writing – original draft. HD’A: Data curation, Formal analysis, Investigation, Writing – original draft. SA: Writing – review & editing. SC: Writing – review & editing. JMac: Writing – review & editing. AP: Writing – review & editing. MR: Writing – review & editing. MT: Writing – review & editing. JMag: Writing – review & editing. MoG: Supervision, Writing – original draft, Writing – review & editing.
